# Integrated multi-platform metabolomics reveals fatty acid-mediated inflammatory signatures in pretibial myxedema

**DOI:** 10.3389/fendo.2026.1734953

**Published:** 2026-01-28

**Authors:** Jiayi Cai, Li Zhang, Jie Zheng, Meng Pan, Xia Li, Xiaoqing Zhao, Shuoting Wang, Zirou Shang, Han Cao, Xiaoying Chen

**Affiliations:** Department of Dermatology, Ruijin Hospital, School of Medicine, Shanghai Jiao Tong University, Shanghai, China

**Keywords:** fatty acid metabolism, fibroblast activation, pretibial myxedema, spatial metabolomics, untargeted metabolomics

## Abstract

**Context:**

Pretibial myxedema (PTM) is a refractory autoimmune dermopathy associated with Graves’ disease. Although metabolic dysregulation has been recognized in thyroid-associated disorders, the metabolic profile and its functional role in PTM remain unclear.

**Objective:**

To characterize the metabolic landscape of PTM lesions and explore the contribution of fatty acids to fibroblast dysfunction and inflammation.

**Methods:**

We performed untargeted metabolomic profiling of PTM skin lesions and healthy controls using LC-MS and GC-MS, integrated with spatial metabolomics to localize metabolic changes. Functional assays were conducted by stimulating human foreskin fibroblasts (HFFs) with palmitic acid (PA) and oleic acid (OA), followed by RNA sequencing, cytokine assays, and immunohistochemistry.

**Results:**

PTM lesions exhibited substantial metabolic dysregulation, including accumulation of fatty acids and elevated tricarboxylic acid cycle intermediates. Spatial metabolomics confirmed pronounced lipid deposition in the dermis, the primary site of PTM pathology. RNA-seq of fibroblasts stimulated with PA and OA revealed enrichment of inflammatory pathways, including IL-17 and NF-κB signaling, and marked upregulation of IL-8 (CXCL8). Fatty acid stimulation induced robust IL-8 secretion, consistent with increased IL-8 expression in PTM lesions. Moreover, PA promoted α-SMA expression in fibroblasts, suggesting induction of myofibroblast differentiation.

**Conclusions:**

Our findings demonstrate that dermal fatty acid accumulation in PTM may contribute to fibroblast-mediated inflammation and fibrosis. This study provides novel insights into the metabolic-immunologic interface underlying PTM pathogenesis.

## Introduction

Pretibial myxedema (PTM) is a refractory autoimmune skin disease that, along with thyroid-associated ophthalmopathy (TAO), represents a common extrathyroidal complication of Graves’ disease (GD). Typical lesions are waxy, discolored indurations or nonpitting edema on the pretibial region, significantly impairing patients’ quality of life (1). Although the pathogenesis of PTM remains unclear, dermal fibroblasts are considered key effector cells regulated by inflammatory cytokines, chemokines, and metabolites (2). In GD, pro-inflammatory mediators from immune cells and fibroblasts act through paracrine and autocrine mechanisms to activate fibroblast, increasing collagen synthesis and hyaluronic acid secretion. These fibroblast-driven processes result in diffuse, nonpitting edema and nodules in PTM, and contribute to exophthalmos and diplopia in TAO (3).

Metabolism has been widely recognized as an important regulator of immune cell differentiation and activation, thereby influencing autoimmune diseases progression (4). Metabolic reprogramming is not only a characteristic of tumor cells but also participates in regulating immune cell activity and chronic inflammatory states (5, 6). In particular, dysregulated fatty acid metabolism can drive pro-inflammatory cytokines. Multiple studies have confirmed that fatty acids, including palmitic and oleic acids, have been shown to induce the expression of pro-inflammatory mediators such as IL-8 by activating the NF-κB pathway and ROS accumulation, thereby sustaining inflammatory responses (7–9). In GD and related conditions, IL-8 has also been implicated as a mediator of chronic, localized tissue inflammation and a potential therapeutic target (10, 11). Abnormal serum lipid profiles have been reported in patients with GD (12). In TAO, disturbances in triglyceride and cholesterol metabolism within orbital adipose tissue have been associated with endoplasmic reticulum stress and adipose tissue inflammation (13). However, the metabolic profile of local skin in PTM, and its potential role in regulating fibroblast activity, remain poorly characterized.

In this study, we conducted untargeted metabolomic profiling using LC−MS and GC−MS to analyze pretibial skin lesions from patients with PTM compared to healthy controls, systematically mapping metabolic alterations. PTM lesions exhibited increased fatty acid metabolism and accumulation of tricarboxylic acid (TCA) cycle intermediates. Spatial metabolomics further confirmed the dermal localization of fatty acid accumulation. *In vitro*, exposure of human foreskin fibroblasts (HFFs) to elevated concentrations palmitic acid (PA) and oleic acid (OA) increased IL-8 expression, suggesting that dysregulated fatty acid metabolism may contribute to PTM pathogenesis by sustaining a pro-inflammatory microenvironment. These findings support a link between aberrant lipid metabolism and fibroblast-mediated inflammation in PTM, and highlight potential metabolic targets for therapeutic intervention.

## Materials and methods

### Human skin tissue samples

Skin samples were collected from 8 patients diagnosed with PTM and 9 healthy controls undergoing cosmetic surgery (**Supplementary Table S1**). Skin biopsies were strictly collected from the pretibial region. All PTM patients had a documented history of GD and were diagnosed by skin histopathology. There were no significant differences in sex or body mass index (BMI) between the two groups. This prospective study was approved by the institutional ethics committee. Informed consent was obtained from all participants.

### Untargeted metabolomics analysis

Skin tissues were processed for LC-MS and GC-MS based untargeted metabolomics as previously described (14, 15). Briefly, tissues were homogenized in methanol (1 mg:50 μL), followed by centrifugation (12, 000 × g, 15 min, 4°C). Supernatants were aliquoted for LC-MS or GC-MS analysis. For LC-MS, samples were mixed with cold methanol containing internal standards; for GC-MS, samples were dried under nitrogen, derivatized with methoxamine hydrochloride and MSTFA, and analyzed under standard chromatographic conditions. Raw data were processed using MassHunter and XCMS-Online for peak detection, alignment, normalization, and missing value imputation. Metabolite identification was based on NIST and HMDB databases. Significant metabolites were defined by *p* ≤ 0.05 and |log_2_FC| ≥ 0.5, and pathway enrichment was performed using MetaboAnalyst 6.0.

### Spatial metabolomics analysis

Frozen tissues were thawed gradually, embedded in Cryo-Gel, sectioned at 20 μm, and mounted on Superfrost Plus slides. One section underwent H&E staining; others were vacuum-dried (~30 min). DESI-MSI was performed in positive and negative ion modes using acetonitrile/water (80:20, v/v) at 1.5 μL/min, with 20 μm spatial resolution, m/z 70–1200, and resolution of 20, 000. Sebaceous gland regions were excluded, and ion images were reconstructed using MassLynx and HDI software.

### Cell culture

HFFs (Zhong Qiao Xin Zhou Biotechnology, Cat# ZQ0450, RRID: CVCL_3285) were cultured in F12/DMEM (Gibco, USA) supplemented with 10% fetal bovine serum (FBS) and 1% penicillin-streptomycin at 37°C in a humidified incubator with 5% CO_2_. When cell confluence reached 70–80%, the medium was replaced, and cells were treated with 200 μM OA or PA for 48 h.

### RNA-seq analysis

Total RNA from HFFs was extracted using TRIzol reagent (Invitrogen) and used to prepare strand-specific RNA-seq libraries for sequencing on the Illumina platform. Clean reads were aligned using HISAT2, and differentially expressed genes (|log_2_FoldChange| > 1, adjusted *p* ≤ 0.05) were identified with DESeq2. Functional enrichment was performed using the ClusterProfiler R package, including GO analysis of biological processes, cellular components, and molecular functions, as well as KEGG pathway analysis, with terms or pathways showing FDR < 0.05 considered significant.

### Cytokine detection

After 48 h of fatty acid treatment, cell culture supernatants were collected for cytokine quantification using a Multi-Analyte Flow Assay Kit (Dake Precision Technology, Shenzhen, China), following the manufacturer’s instructions. Flow cytometric analysis was performed on a BD FACSCanto™ system, and data were processed using LEGENDplex™ software.

### Western blot

Cells were lysed in RIPA buffer with protease inhibitors at 4°C for 15 min, then centrifuged at 12, 000 × g for 15 min. Protein concentration was normalized, and samples were separated via SDS-PAGE and transferred to PVDF membranes. After blocking for 15 min, membranes were incubated overnight with primary antibodies: α-SMA (Thermo Fisher Scientific, Cat# 14-9760-80, RRID: AB_2572995), ADFP (Invitrogen, Cat# PA1-16972, RRID: AB_2223607), and tubulin (Proteintech, Cat# HRP-66031, RRID: AB_2687491). After washing, membranes were incubated with HRP-conjugated secondary antibodies, anti-mouse IgG (Epizyme Biomedical Technology, Cat# LF101, RRID: AB_3083706) and anti-rabbit IgG (ABCAM, Cat# Ab6721, RRID: AB_955447), for 1 h at room temperature. Bands were visualized using an ECL detection kit.

### Immunohistochemistry staining

Paraffin-embedded tissue sections were deparaffinized, rehydrated, and subjected to antigen retrieval. Endogenous peroxidase was blocked with 3% H_2_O_2_, followed by blocking with normal goat serum. Sections were incubated overnight at 4°C with anti-IL-8 antibody (Thermo Fisher Scientific, Cat# PA5-79114, RRID: AB_2746230), then with HRP-conjugated secondary antibody, and developed using DAB. Nuclei were counterstained with hematoxylin. Positive and negative controls were included, and staining was evaluated by light microscopy. The percentage of positive cells was calculated by dividing the number of positively stained cells by the total number of cells in four randomly selected high-power fields (HPFs) per section.

## Results

### Untargeted metabolomics delineates global metabolic alterations in PTM lesions

**Figure 1:**
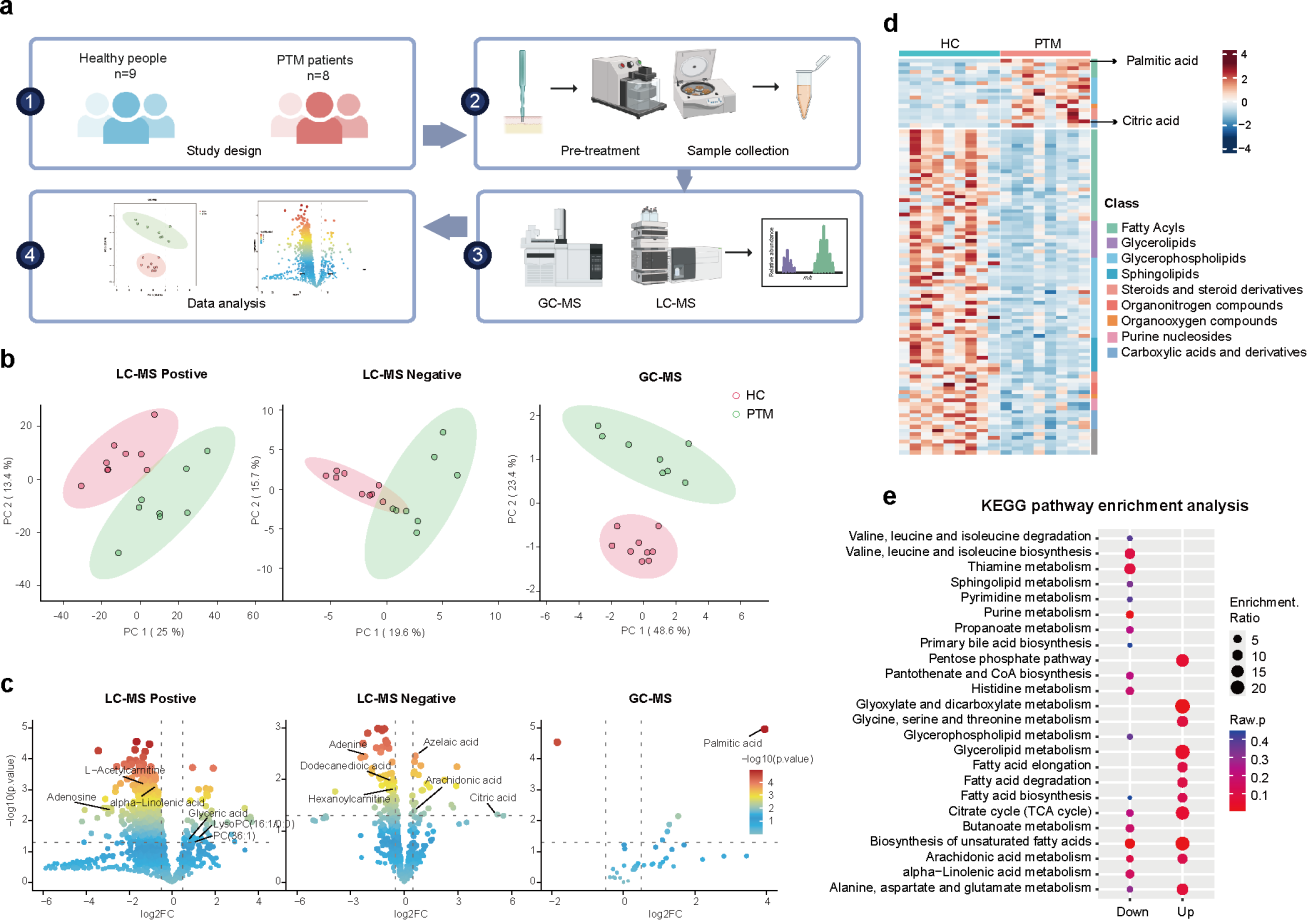
Dual-platform metabolomic analysis reveals metabolic differences between PTM lesions and healthy skin. **(a)** Schematic diagram of the experimental workflow. **(b)** PCA plots of metabolomic profiles from PTM patients and healthy controls based on LC-MS and GC-MS platforms. **(c)** Volcano plots showing differential metabolites identified on each platform, with representative upregulated and downregulated metabolites labeled. Horizontal dashed line: *p* = 0.05. Vertical dashed lines: |log_2_ fold change| = 0.5. **(d)** Heatmap of 106 differential metabolites identified across both platforms. **(e)** KEGG pathway enrichment analysis.

To characterize the metabolic profiles of PTM skin lesions, we performed untargeted metabolomics analyses using both LC-MS and GC-MS platforms on pretibial skin from patients with PTM and healthy controls (**Figure 1a**). Principal component analysis (PCA) showed distinct clustering of PTM and control groups across LC-MS positive/negative ion modes and the GC-MS platform suggesting significant metabolic divergence in PTM lesions (**Figure 1b**).

LC-MS in positive ion mode identified 53 significantly upregulated and 817 significantly downregulated metabolites. Upregulated metabolites included glyceric acid (fold change [FC] = 1.79), LysoPC (16:1/0:0) (FC = 2.26), and PC (36:1) (FC = 2.00). Downregulated metabolites mainly involved lipids and carbohydrate-related compounds such as α-linolenic acid (FC = 0.63), adenosine (FC = 0.14), and L-acetylcarnitine (FC = 0.41). In the negative ion mode, 22 upregulated metabolites were detected, including citric acid (FC = 36.9), azelaic acid (FC = 1.53), and arachidonic acid (FC = 1.47), alongside 108 downregulated metabolites such as adenine (FC = 0.21), dodecanedioic acid (FC = 0.62), and hexanoylcarnitine (FC = 0.67). GC-MS analysis identified 9 upregulated and 1 downregulated metabolites. Notably, PA was markedly elevated (FC = 15.47) (**Figure 1c**), highlighting PA as a markedly altered metabolite in PTM lesions. By integrating results from both platforms, we identified a total of 107 potential biomarkers, including significantly upregulated citric acid, PA, and LysoPCs, as well as downregulated L-acetylcarnitine, docosatrienoic acid, and arachidic acid (**Figure 1d**, **Supplementary Table S2**).

KEGG pathway enrichment analysis revealed that both upregulated and downregulated metabolites were significantly enriched in key energy and lipid metabolic pathways, including the biosynthesis of unsaturated fatty acid, citrate cycle (TCA cycle), and arachidonic acid metabolism. Additionally, pathways related to fatty acid synthesis, elongation, and degradation were altered (**Figure 1e**). These findings indicate a pronounced dysregulation of fatty acid metabolism and TCA cycle activity in PTM lesions.

### Spatial metabolomics reveals dermal fatty acid–driven metabolic dysregulation in PTM

To investigate regional metabolic alterations in PTM, we applied DESI-MSI to pretibial skin samples from a PTM patient and a healthy control, focusing on fibroblast-rich dermal compartments. To minimize variability due to structural heterogeneity, we excluded areas containing sebaceous glands and restricted the spatial analysis to matched dermal regions, thereby reducing bias from lipid-dense structures.

**Figure 2:**
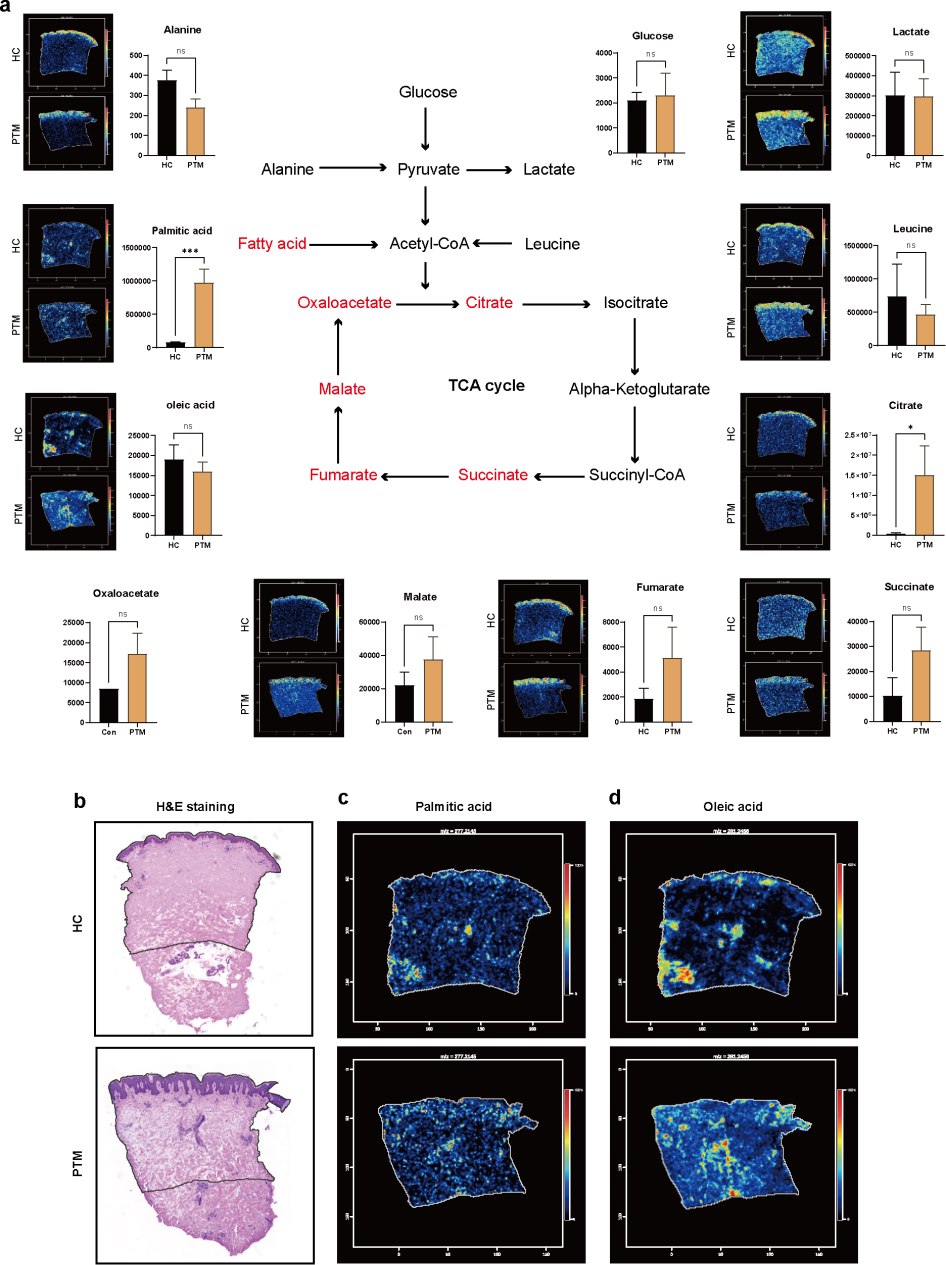
Integrated analysis of conventional and spatial metabolomics. **(a)** Schematic illustration of the TCA cycle and its metabolic links to carbohydrate, lipid, and amino acid metabolism, with detected metabolites in PTM lesions annotated. PTM (*n* = 8), HC (*n* = 9), error bars indicate ± SEM. **(b)** H&E staining images of PTM lesions and healthy control skin **(c)** Ion imaging of OA in negative ion mode. **(d)** Ion imaging of PA in negative ion mode. OA, oleic acid; PA, palmitic acid; Statistical analysis: Student’s t-test; *p < 0.05, ***p < 0.001; ns, not significant.

We selected representative metabolites from the TCA cycle, fatty acid metabolism, glycolysis and amino acid metabolism pathways, and visualized their trends through histograms of conventional metabolism and spatial metabolomic ion images (**Figure 2a**). The results showed a general upregulation of TCA cycle intermediates, with citric acid exhibiting a particularly strong increase (FC = 36.9, *p* < 0.05). Other intermediates, including cis-aconitate, malic acid, and oxaloacetate, did not reach statistical significance but displayed a consistent upward trend. These findings indicate a profound dysregulation of mitochondrial homeostasis in PTM lesions. In contrast, no significant changes were observed in glycolytic or amino acid metabolites, including glucose, lactate, and most amino acids. PA was significantly elevated in conventional and spatial metabolism, while OA was significantly upregulated in spatial metabolism.

Given that PTM is characterized by fibroblast activation predominantly within the dermis, we co-registered DESI-MSI ion images with matched H&E-stained sections to spatially resolve metabolic alterations in the dermal compartment (**Figure 2b**). Dermal fatty acid signals were markedly elevated, with focal enrichment patterns most pronounced for PA and OA (**Figures 2c, d**). These findings suggest a dermal lipid-accumulation signature in PTM lesions, with a potential association with fibroblast activation.

### Transcriptomic profiling reveals PTM-associated fatty acids activate pro-inflammatory programs in fibroblasts

To investigate the pro-inflammatory effects of fatty acids on fibroblasts, we treated HFFs with OA and PA, both of which were identified as elevated in PTM lesions, and performed transcriptomic profiling using RNA-seq.

**Figure 3:**
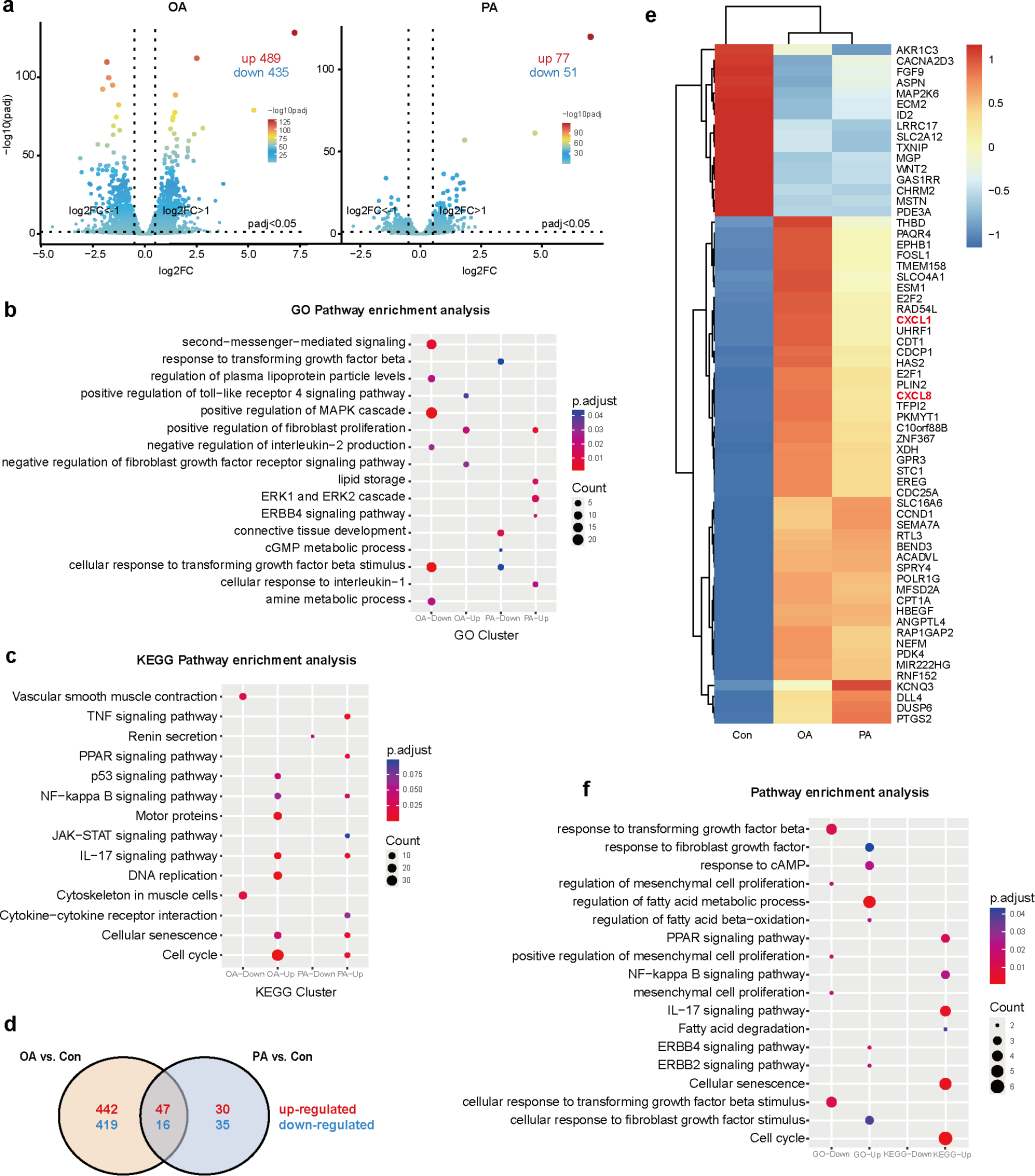
Transcriptomic profiling of fibroblasts following OA and PA treatments. **(a)** Volcano plots showing DEGs in fibroblasts treated with OA or PA compared to vehicles. **(b)** GO enrichment analysis of DEGs after OA and PA treatment. **(c)** KEGG pathway enrichment analysis of DEGs after OA and PA treatment. **(d)** Venn diagram showing overlapping up- and downregulated genes between OA and PA treatments. **(e)** Heatmap of 63 shared DEGs between the OA and PA groups. **(f)** GO and KEGG enrichment analysis of commonly regulated genes. DEGs, differentially expressed genes.

Compared with the control group, OA treatment led to 489 upregulated genes and 435 downregulated genes, whereas PA treatment resulted in 77 upregulated genes and 51 downregulated genes (**Figure 3a**). GO analysis showed that OA-upregulated genes were enriched in Toll-like receptor signaling and the positive regulation of fibroblast proliferation. PA-upregulated genes were enriched in the positive regulation of fibroblast proliferation, the ERK1/ERK2 cascade, and the ERBB4 signaling pathway (**Figure 3b**). KEGG pathway analysis revealed an enrichment of inflammation-related pathways. Following OA treatment, upregulated genes were enriched in the IL-17, p53, and NF-κB signaling pathways. Following PA treatment, upregulated genes were enriched in the TNF, IL-17, and JAK-STAT signaling pathways (**Figure 3c**). These results suggest that both OA and PA treatments activate inflammation-related signaling pathways in fibroblasts and may influence their differentiation.

Intersection analysis identified 47 commonly upregulated and 16 downregulated genes (**Figure 3d**). Two key pro-inflammatory chemokines, *CXCL1* and *CXCL8*, were consistently elevated (**Figure 3e**), implicating that fatty acids may trigger fibroblast-mediated inflammatory responses via these mediators. Pathway enrichment analysis of the shared upregulated genes revealed significant involvement of the IL-17 and NF-κB signaling pathways, both of which play central roles in inflammatory regulation (**Figure 3f**). These findings suggest that fatty acid accumulation in PTM lesions may promote local immune activation by stimulating inflammatory gene expression in fibroblasts through classical pro-inflammatory signaling pathways.

### *In vitro* evidence linking dermal fatty acid accumulation in PTM to sustained inflammation and fibroblast differentiation

**Figure 4:**
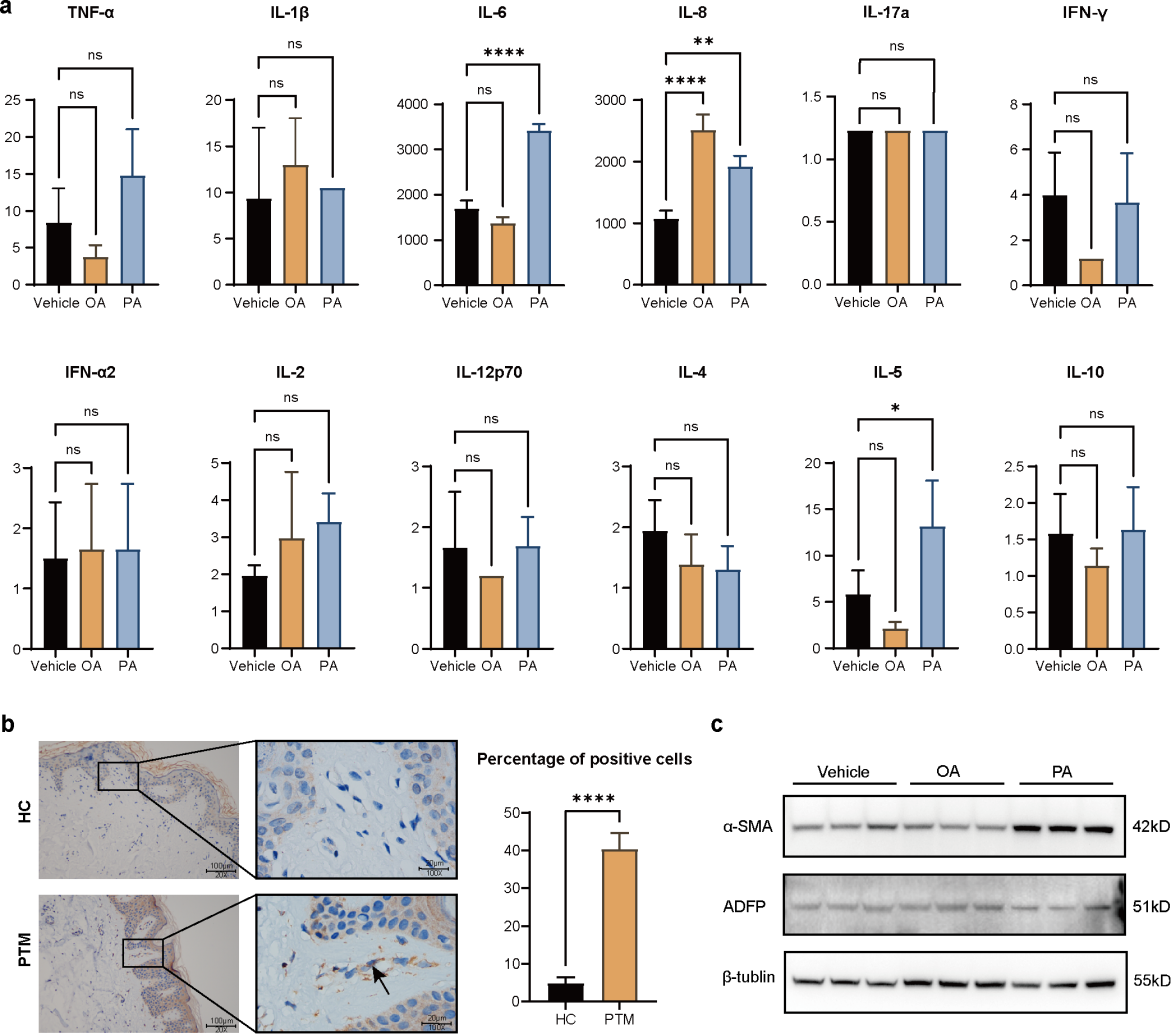
OA and PA sustain inflammation in PTM lesions by inducing IL-8 secretion from fibroblasts **(a)** Cytokine expression in fibroblasts following 48-hour treatment with Vehicle, OA or PA. *n* = 3; error bars indicate ± SEM. **(b)** Representative immunohistochemical staining of IL-8 in healthy control and PTM skin sections. Arrows indicate representative IL-8-positive cells. Scale bars = 100 μm (overview) and 20 μm (high magnification). Quantification is presented as the percentage of IL-8 positive cells per high-power field (HPF). PTM (*n* = 6), HC (*n* = 5); error bars indicate ± SEM. **(c)** Western blot analysis of α-SMA and ADFP expression in fibroblasts after treatments. Statistical analysis: Student’s t-test (two-group comparisons) or one-way ANOVA (multiple-group comparisons); *p < 0.05, **p < 0.01, ****p < 0.0001; ns, not significant.

Based on transcriptomic analysis showing activation of inflammatory pathways in fibroblasts after OA and PA exposure, we further examined their effects on cytokine secretion and fibroblast phenotype. HFFs were treated with OA or PA, and inflammatory cytokines in the culture supernatant were subsequently quantified. Both OA and PA treatment significantly increased IL-8 secretion, whereas PA also significantly increased IL-6 levels (**Figure 4a**). To assess disease relevance, IL-8 expression in skin sections from PTM patients and healthy controls was examined by immunohistochemistry. The percentage of IL-8 positive cells was significantly higher in PTM lesions than in healthy controls (**Figure 4b**). These findings suggest that altered fatty acid metabolism may play a role in sustaining local inflammation in PTM skin by promoting fibroblast-derived IL-8 production.

We also examined the effect of dysregulated fatty acid metabolism on fibroblast differentiation by assessing the expression of two classical differentiation markers: α-smooth muscle actin (α-SMA) for myofibroblasts (16), and adipose differentiation-related protein (ADFP) for adipocytes (17, 18) Western blot analysis revealed that ADFP expression remained unchanged following OA or PA treatment; however, PA stimulation significantly upregulated α-SMA expression, suggesting a fibroblast-to-myofibroblast phenotypic transition and potentially contributing to tissue remodeling and fibrosis. (**Figure 4c**).

To further validate the physiological relevance of these findings, we repeated the key experiments using primary dermal fibroblasts isolated from adult skin. Consistent with the observations in HFFs, PA treatment significantly upregulated the secretion of IL-8 and IL-6 (**Supplementary Figure S1a**) and increased the expression of α-SMA (**Supplementary Figure S1b**) in primary fibroblasts.

## Discussion

This study employed untargeted metabolomics integrating LC-MS and GC-MS platforms to characterize metabolic alterations in pretibial skin lesions from PTM patients and matched healthy controls. PTM lesions showed significant accumulation of fatty acids and elevated TCA cycle intermediates, while glycolytic metabolites and amino acids remained largely unchanged, indicating a metabolic shift favoring fatty acid-driven energy metabolism. Spatial metabolomics further localized fatty acid enrichment, especially palmitic and oleic acids, to fibroblast-rich dermal compartments, supporting the concept that local lipid accumulation contributes to metabolic dysregulation in PTM.

Accumulation of long-chain fatty acids is a recognized metabolic hallmark in various inflammatory skin diseases (19, 20). In GD and its complications such as TAO, systemic lipid metabolic abnormalities have been reported (21), and impaired cholesterol homeostasis and increased free fatty acids are associated with the severity of TAO (22, 23). Our study focuses on the local metabolic microenvironment. The specific enrichment of PA in the dermis implies that tissue-resident accumulation might be a critical factor in establishing the local pathogenic niche, potentially distinct from systemic serum levels. Free fatty acids (FFAs) serve as substrates for β-oxidation, producing acetyl-CoA that fuels the TCA cycle to support mitochondrial ATP generation, which may enhance cellular adaptation to inflammatory stress (24, 25). Our data showed significant upregulation of FFAs including PA and OA, alongside increased TCA cycle intermediates in PTM lesions. Spatial metabolomics revealed marked dermal enrichment of these FFAs, while OA showed accumulation in the dermis despite nonsignificant changes in bulk tissue analysis, likely reflecting the superior spatial resolution of this technique (26, 27). Taken together, these findings suggest that aberrant fatty acid metabolism, rather than glycolysis or amino acid metabolism, is closely linked to the observed dysregulation of the TCA cycle in PTM, indicating a fatty acid-driven metabolic dysregulation in lesional skin.

Both PA, a saturated fatty acid, and OA, a monounsaturated fatty acid, induced IL-8 expression in dermal fibroblasts, establishing a mechanistic link between fatty acid accumulation and local inflammation in PTM. Notably, PA also significantly increased IL-6 secretion, whereas OA did not, reflecting the stronger pro-inflammatory capacity of saturated fatty acids.PA also significantly upregulated α-SMA expression, indicative of fibroblast-to-myofibroblast differentiation and potential promotion of tissue remodeling. OA showed no significant effect on α-SMA levels, suggesting a lesser role in fibrotic processes. Prior studies have documented PA-induced fibrosis and α-SMA upregulation in various organs, while unsaturated fatty acids may mitigate fibrotic responses (28–30). These results highlight a dual pathological role of PA in PTM by driving both inflammation and fibroblast activation.

Our results suggest a divergent pathogenic model for different lipid species. While both PA and OA contribute to the inflammatory microenvironment, they appear to utilize distinct signaling pathways. Saturated fatty acids (PA) significantly enriched TNF and JAK-STAT signaling pathways in our transcriptomic analysis, which are known drivers of myofibroblast differentiation (31, 32). This likely explains why PA specifically induced α-SMA expression and fibrosis. In contrast, unsaturated fatty acids (OA) primarily activated the IL-17 signaling pathway, fueling inflammation without triggering the myofibroblast transition. Instead of a generalized “fatty acid effect, “ this distinction suggests that the specific accumulation of saturated lipids like PA is the critical driver for the induration (fibrosis) characteristic of PTM.

In GD and its complications, IL-8 is considered an important inflammatory mediator. In TAO, IL-8 has been shown to be elevated in both serum and conjunctival epithelium, and is thought to contribute to the persistence of local inflammation (11, 33). IL-8 co-localizes with extracellular matrix-associated molecules and may promote tissue remodeling (34). Moreover, IL-8 may exacerbate local tissue damage by enhancing lymphocyte infiltration in the thyroid and amplifying fibroblast inflammatory responses in TAO lesions (35). Our findings extend these observations by demonstrating increased IL-8 expression in PTM skin, which may be driven by local fatty acid accumulation, linking metabolic dysregulation to immune activation in PTM pathogenesis.

Interestingly, we did not observe a linear correlation between PA abundance and clinical severity scores within the PTM cohort. This likely reflects the temporal uncoupling of the disease: metabolic accumulation may act as an initiating trigger. In chronic stages, metabolic levels may plateau while structural damage persists, explaining the lack of linearity in established lesions.

There are limitations to this study. First, the small number of spatial metabolomics samples, due to the rarity of PTM and difficulty in obtaining pretibial skin, limits the generalizability of our spatial findings and may partly explain discrepancies with bulk metabolomics data. The DESI-MSI data should be interpreted as semi-quantitative visualizations of lipid localization. Given the technical constraints (e.g., lack of absolute quantification standards) and the small cohort size, these results highlight spatial enrichment patterns but do not support precise quantitative comparisons typically achieved with LC-MS. Second, the absence of a validated animal model prevented *in vivo* functional validation, limiting direct demonstration of causal relationships between fatty acid accumulation, inflammation, and metabolic dysregulation. Third, while we identified TCA intermediate accumulation, real-time metabolic flux analysis was not performed, limiting our ability to definitively determine metabolic turnover rates. Future studies should increase sample sizes, integrate multi-omics, and establish relevant models to delineate the mechanistic role of lipid metabolism in PTM.

In conclusion, this study depicts the metabolic profile of pretibial myxedema by bulk metabolomics and spatial metabolomics, with accumulation of long-chain fatty acids such as palmitic acid and oleic acid, and significant accumulation of TCA cycle intermediates. Elevated fatty acids induce fibroblasts to secrete pro-inflammatory factors, especially IL-8, which may help maintain the chronic inflammatory state at the lesion. Our findings suggest a potential role of immunometabolism in PTM pathogenesis and raise the possibility that modulation of fatty acid metabolism and related inflammatory pathways could represent a therapeutic avenue.

## Data Availability

The original contributions presented in this study are publicly available. The data are available in the NCBI Gene Expression Omnibus (GEO) database under accession number GSE317276.
