# Supplementary figures and images for: Integrated multi-platform metabolomics reveals fatty acid-mediated inflammatory signatures in pretibial myxedema
Source: Front Endocrinol (Lausanne). 2026 Jan 28;17:1734953. doi: 10.3389/fendo.2026.1734953 (PMC12890676; doi:10.3389/fendo.2026.1734953)

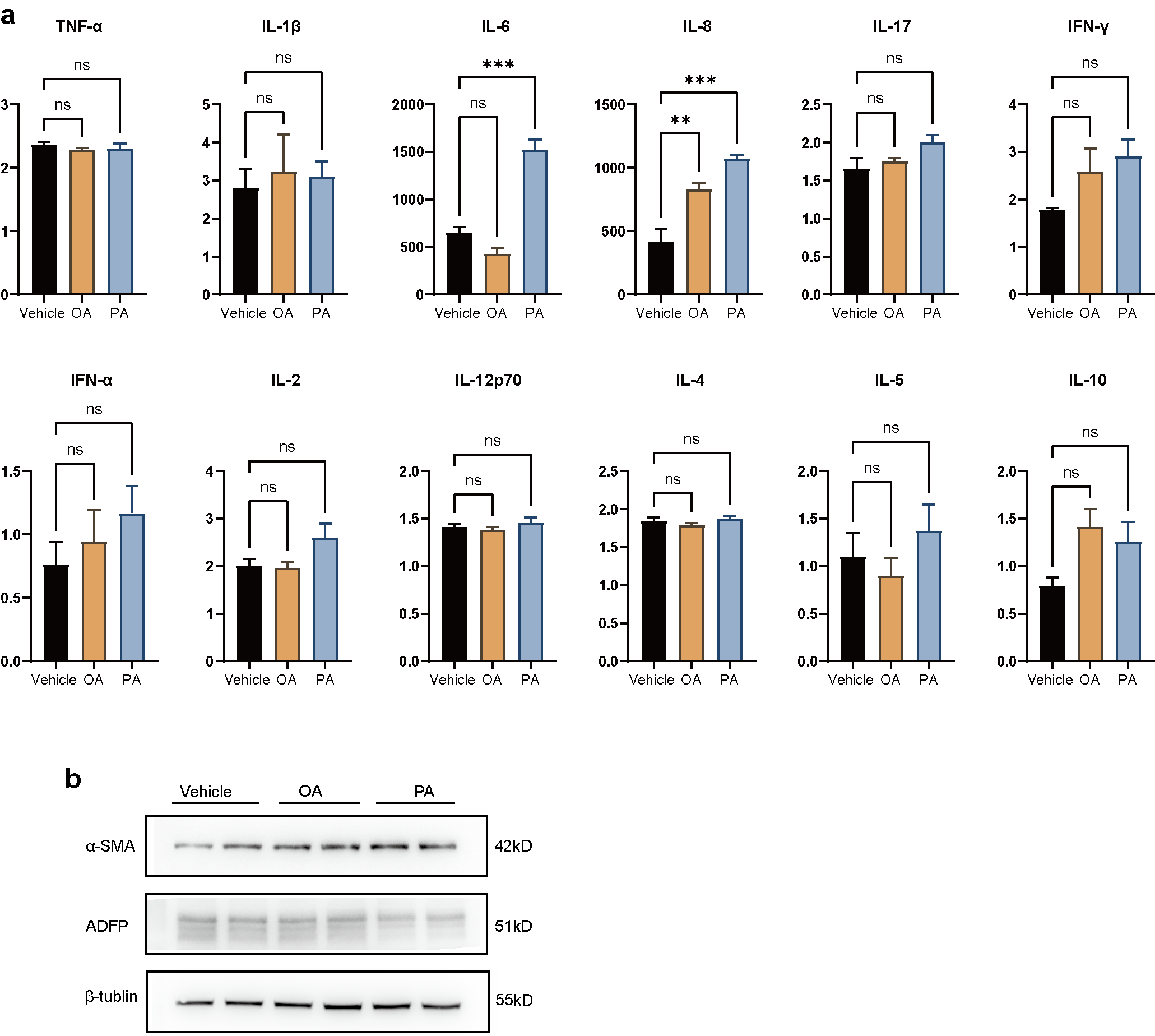

Supplement: Supplementary file 1 [file Image1.tif]
