# Supplementary material for: Integrated multi-platform metabolomics reveals fatty acid-mediated inflammatory signatures in pretibial myxedema
Source: Front Endocrinol (Lausanne). 2026 Jan 28;17:1734953. doi: 10.3389/fendo.2026.1734953 (PMC12890676; doi:10.3389/fendo.2026.1734953)
Supplement: Supplementary file 2 [file Table1.docx]

**SUPPLEMENTARY MATERIALS**

Supplementary Table S1. Clinical information for PTM patients

| patient no. | sex | age at surgery | PTM duration to surgery (months) | skin dermal thickness in the lesions (mm) | smoking status | TRAb preop | TSI preop |
| --- | --- | --- | --- | --- | --- | --- | --- |
| PTM-01 | f | 54 | 24 | 6.6 | 0 | >40 | 29.6 |
| PTM-02 | f | 49 | 6 | 2.7 | 0 | >40 | >40 |
| PTM-03 | f | 35 | 24 | 8.1 | 0 | >40 | >40 |
| PTM-04 | m | 36 | 12 | 13.7 | 2 | >40 | / |
| PTM-05 | f | 42 | 2 | 7.1 | 0 | 17,87 | 13.5 |
| PTM-06 | m | 22 | 18 | 7.7 | 0 | 8.1 | 1.76 |
| PTM-07 | m | 36 | 6 | 6.7 | 0 | >40 | >40 |
| PTM-08 | m | 59 | 9 | 5.5 | 2 | 9.98 | 3.6 |

Supplementary Table S2. Differential metabolites from both platforms

| Metabolite | M/Z | RT (min) | P-value | FC |
| --- | --- | --- | --- | --- |
| Palmitic acid | 274.2754 | 8.39 | 1.09E-05 | 15.46912 |
| Androsterone sulfate | 369.1732 | 7.95 | 0.005638 | 7.68607 |
| Citric acid | 191.0212 | 1.71 | 0.047436 | 36.90639 |
| Testosterone sulfate | 367.1581 | 7.45 | 0.013275 | 7.504967 |
| PI(18:0/20:4(5Z,8Z,11Z,14Z)) | 885.5464 | 14.01 | 0.036296 | 2.562748 |
| Azelaic acid | 187.0987 | 5.81 | 0.003506 | 1.534116 |
| LysoPC(P-18:0/0:0) | 552.3642 | 14.22 | 0.005361 | 1.902143 |
| Arachidonic acid | 303.2326 | 18.27 | 0.040297 | 1.474182 |
| PC(32:2) | 788.5411 | 6.87 | 0.030803 | 6.223679 |
| Sebacic acid | 261.135 | 5.5 | 0.008108 | 1.735103 |
| Glycoursodeoxycholic acid | 448.3049 | 9.32 | 0.010135 | 4.746933 |
| PC(22:4(7Z,10Z,13Z,16Z)/16:0) | 832.5799 | 15.35 | 0.010834 | 1.89833 |
| gamma-Glutamylhistidine | 302.1447 | 13.81 | 0.024715 | 1.638604 |
| PE(15:0/24:1(15Z)) | 810.5996 | 19.62 | 0.047404 | 2.923677 |
| Glyceric acid | 124.0597 | 1.66 | 0.043325 | 1.785927 |
| PC(36:1) | 810.5979 | 11.32 | 0.04718 | 2.002071 |
| LysoPC(16:1/0:0) | 494.3236 | 11.35 | 0.037902 | 2.25822 |
| Valerylcarnitine | 246.1703 | 4.42 | 0.00469 | 3.572129 |
| L-Acetylcarnitine | 221.1516 | 9.03 | 0.000707 | 0.409925 |
| Docosatrienoic acid | 352.3186 | 11.36 | 0.000884 | 0.441578 |
| Stearic acid | 302.3065 | 10.12 | 0.003121 | 0.548645 |
| Arachidic acid | 330.338 | 11.36 | 0.007101 | 0.597096 |
| Adenosine | 268.1044 | 3.02 | 0.00485 | 0.141417 |
| SM(d18:1/22:0) | 787.6672 | 19.52 | 0.003791 | 0.11126 |
| Dodecanoic acid | 218.212 | 5.76 | 0.01072 | 0.462198 |
| Cer(d18:1/14:0) | 510.4872 | 11.53 | 0.003663 | 0.523492 |
| Cer(d18:0/14:0) | 512.503 | 19.86 | 0.005214 | 0.513316 |
| MG(16:0/0:0/0:0) | 331.2839 | 18.24 | 0.00148 | 0.431516 |
| SM(d18:0/24:1(15Z)) | 837.6795 | 15.35 | 0.000853 | 0.452956 |
| Glutamylthreonine | 249.1104 | 5.81 | 0.000937 | 0.412616 |
| alpha-Linolenic acid | 296.2564 | 8.39 | 0.000713 | 0.532295 |
| Cer(d18:1/16:0) | 538.5188 | 11.58 | 0.003332 | 0.548207 |
| PG(16:0/16:0) | 723.5127 | 23.88 | 0.000618 | 0.524709 |
| SM(d17:1/24:0) | 801.6833 | 15.35 | 0.003703 | 0.485825 |
| SM(d18:1/26:0) | 843.7293 | 15.35 | 0.004394 | 0.578237 |
| Pentadecanoic acid | 260.2586 | 7.8 | 0.004797 | 0.546786 |
| SM(d18:1/24:0) | 815.6984 | 14.5 | 0.000175 | 0.229452 |
| PC(O-44:5) | 895.7344 | 24.99 | 0.010561 | 0.054287 |
| 3-Hydroxybutyrylcarnitine | 248.151 | 1.07 | 0.000138 | 0.342445 |
| Butyrylcarnitine | 249.1829 | 11.82 | 0.002245 | 0.481204 |
| Oleic acid | 300.2894 | 8.9 | 0.002493 | 0.586325 |
| MG(18:2(9Z,12Z)/0:0/0:0) | 377.263 | 10.24 | 0.008732 | 0.565327 |
| SM(d18:1/24:1) | 835.6647 | 19.58 | 0.000409 | 0.272218 |
| DG(15:0/16:1(9Z)/0:0) | 570.5081 | 11.56 | 0.009774 | 0.596457 |
| LysoPA(i-14:0/0:0) | 400.2485 | 10.24 | 0.011469 | 0.592083 |
| Succinic acid | 136.0616 | 3.02 | 0.005933 | 0.223501 |
| Tetradecanoylcarnitine | 394.2894 | 10.23 | 0.004291 | 0.590042 |
| FAHFA(18:0/6-O-16:0) | 556.529 | 11.58 | 0.010411 | 0.576631 |
| Lithocholic acid | 394.33 | 13.41 | 0.000181 | 0.347501 |
| PC(36:0) | 790.6279 | 19.61 | 0.003377 | 0.246678 |
| PC(O-44:6) | 893.719 | 24.99 | 0.049215 | 0.057959 |
| 8-Isoprostane | 298.347 | 14.52 | 0.00097 | 0.480954 |
| Tetracosanoic acid | 386.4002 | 17.52 | 0.016033 | 0.358224 |
| Phosphate | 98.9837 | 10.01 | 0.005713 | 0.543671 |
| DG(LTE4/0:0/i-16:0) | 774.4959 | 10.24 | 0.012395 | 0.506295 |
| Aniline | 94.065 | 2.87 | 0.016231 | 0.627827 |
| 12-HETE | 321.24 | 11.23 | 0.013803 | 0.472705 |
| Leukotriene B4 | 337.2346 | 11.25 | 0.014102 | 0.553043 |
| PC(36:2) | 786.6018 | 19.61 | 0.010379 | 0.195804 |
| TG(14:1(9Z)/14:0/20:3n6) | 816.702 | 15.35 | 0.002382 | 0.58693 |
| PE-NMe(24:1(15Z)/22:2(13Z,16Z)) | 913.7416 | 20.54 | 0.001973 | 0.115215 |
| Urocanic acid | 139.0515 | 3.87 | 0.045884 | 0.306985 |
| Stearoylethanolamide | 328.3209 | 19.22 | 0.035115 | 0.376496 |
| Sphingosine | 322.2717 | 10.64 | 0.0276 | 0.464058 |
| Nervonic acid | 384.3834 | 18.15 | 0.018672 | 0.520125 |
| Cholesteryl acetate | 429.3722 | 24.18 | 0.042337 | 0.496848 |
| DG(TXB2/0:0/a-25:0) | 809.6484 | 15.35 | 0.026285 | 0.483595 |
| LysoPC(20:3/0:0) | 568.3384 | 10.92 | 0.015385 | 0.556537 |
| LysoPE(P-18:0/0:0) | 488.3102 | 15.42 | 0.01897 | 0.587055 |
| Desmosine | 548.2743 | 11.32 | 0.000385 | 0.580279 |
| Glutarylcarnitine | 293.1724 | 6.43 | 0.009995 | 0.6139 |
| 12-Hydroxy-12-octadecanoylcarnitine | 444.3679 | 11.87 | 0.018123 | 0.48826 |
| LysoPE(20:1(11Z)/0:0) | 525.367 | 20.11 | 0.028191 | 0.640811 |
| TG(14:0/20:3n6/14:1(9Z)) | 816.702 | 19.55 | 0.030111 | 0.30388 |
| PC(40:6) | 834.5958 | 19.61 | 0.036261 | 0.242051 |
| L-Valine | 140.0679 | 5.37 | 0.016813 | 0.570655 |
| Leukotriene A4 | 319.2242 | 13.88 | 0.016862 | 0.404345 |
| Thromboxane | 319.2991 | 20.74 | 0.01256 | 0.351065 |
| PE-NMe(15:0/22:2(13Z,16Z)) | 789.6181 | 15.35 | 0.022722 | 0.597126 |
| MG(0:0/17:0/0:0) | 362.3268 | 8.62 | 0.001685 | 0.587141 |
| LysoPE(18:2(9Z,12Z)/0:0) | 500.2751 | 10.62 | 0.012062 | 0.466276 |
| Cholic acid | 426.3199 | 15.35 | 0.018669 | 0.525502 |
| LysoPC(0:0/16:0) | 496.3392 | 11.38 | 0.034619 | 0.374075 |
| 3-Hydroxyhexadecanoylcarnitine | 416.3372 | 10.67 | 0.037893 | 0.352066 |
| 2-Hexenoylcarnitine | 199.0949 | 3.95 | 0.024171 | 0.576562 |
| Phosphorylcholine | 206.0558 | 0.98 | 0.009412 | 0.546499 |
| Thiamine | 282.1395 | 7.54 | 0.003444 | 0.475796 |
| MG(15:0/0:0/0:0) | 339.2503 | 11.18 | 0.02401 | 0.523078 |
| Uridine | 245.0786 | 9.54 | 0.045705 | 0.673647 |
| LysoPS(18:1(9Z)/0:0) | 524.2968 | 12.44 | 0.029985 | 0.565945 |
| Guanosine | 306.0805 | 3.11 | 0.003407 | 0.578286 |
| PS(15:0/18:2(9Z,12Z)) | 763.5163 | 23.9 | 0.026631 | 0.649809 |
| PA(16:1(9Z)/15:0) | 633.4533 | 18.64 | 0.036033 | 0.55922 |
| PS(16:1(9Z)/20:0) | 812.5414 | 19.59 | 0.02878 | 0.403382 |
| Inosine | 291.0704 | 3.1 | 0.0032 | 0.6703 |
| PE(22:1(13Z)/14:0) | 763.6004 | 15.35 | 0.045126 | 0.567518 |
| PA(18:2(9Z,12Z)/15:0) | 681.4471 | 20.72 | 0.029737 | 0.676256 |
| MG(i-15:0/0:0/0:0) | 334.2953 | 8.6 | 0.039134 | 0.643522 |
| Adenine | 134.0478 | 3.01 | 0.003582 | 0.21677 |
| Prostaglandin J2 | 393.2272 | 10.13 | 0.015694 | 0.543292 |
| 2-Heptanone | 173.1187 | 7.17 | 0.043708 | 0.642417 |
| LysoPI(18:1(9Z)/0:0) | 597.3064 | 18.67 | 0.001952 | 0.518957 |
| Glutamylarginine | 362.1659 | 9.59 | 0.038209 | 0.668861 |
| Dodecanedioic acid | 229.1453 | 7.88 | 0.011076 | 0.621731 |
| Hexanoylcarnitine | 258.172 | 6.16 | 0.014523 | 0.678362 |
| MG(PGD1/0:0/0:0) | 487.2869 | 18.67 | 0.01604 | 0.626939 |
| LysoPI(18:2(9Z,12Z)/0:0) | 655.3043 | 16.19 | 0.013482 | 0.612261 |
